# Pegfilgrastim (Neulasta)-induced acute aortitis: a case report

**DOI:** 10.1093/ehjcr/ytaf513

**Published:** 2025-10-08

**Authors:** Pranay Marlecha, Samir Kapadia, Akiva Rosenzveig, Robert Wolff, Aravinda Nanjundappa

**Affiliations:** Sections of Clinical Cardiology and Vascular Medicine Robert and Suzanne Tomsich Department of Cardiovascular Medicine, Cleveland Clinic, Desk J3-5 9500 Euclid Avenue, Cleveland, OH 44195, USA; Sections of Clinical Cardiology and Vascular Medicine Robert and Suzanne Tomsich Department of Cardiovascular Medicine, Cleveland Clinic, Desk J3-5 9500 Euclid Avenue, Cleveland, OH 44195, USA; Sections of Clinical Cardiology and Vascular Medicine Robert and Suzanne Tomsich Department of Cardiovascular Medicine, Cleveland Clinic, Desk J3-5 9500 Euclid Avenue, Cleveland, OH 44195, USA; Department of Medical Oncology, Division of Cancer Medicine, MD Anderson Cancer Centre, 1515 Holcombe Blvd, Houston, TX 77030, USA; Sections of Clinical Cardiology and Vascular Medicine Robert and Suzanne Tomsich Department of Cardiovascular Medicine, Cleveland Clinic, Desk J3-5 9500 Euclid Avenue, Cleveland, OH 44195, USA

**Keywords:** Aortitis, Pegfilgrastim, G-CSF, Neulasta, PET

## Abstract

**Background:**

Granulocyte colony-stimulating factor (G-CSF) and especially pegfilgrastim for its long-lasting effects are commonly used to lower the chances of neutropenia during chemotherapy. However, extremely rarely, it may result in large-vessel vasculitis such as aortitis which is hard to detect and manage.

**Case summary:**

A 59-year-old woman with pancreatic adenocarcinoma on pegfilgrastim developed fever and upper back pain 2 days after administration. Laboratory investigations showed elevated inflammatory markers, but microbiology and autoimmune tests were negative, including tests for tuberculosis, syphilis, and Q fever. Computed tomography (CT) imaging showing periaortic fat stranding and grade II/III FDG uptake in the ascending aorta and arch on the positron emission tomography (PET) scan aided in the diagnosis of acute aortitis. Absence of other possible aetiologies and the close temporal association to pegfilgrastim administration, a diagnosis of G-CSF-induced acute aortitis was made. A multidisciplinary team managed and monitored her conservatively without glucocorticoids, due to her comorbidities. Clinical improvement with normalization of inflammatory markers and repeat PET imaging was seen over several weeks.

**Discussion:**

Granulocyte colony-stimulating factor–induced acute aortitis is a new entity emerging, with possible fatal consequences. This case shows the importance of a thorough diagnostic workup, adept interpretation of PET/CT scans with vasculitis imaging standards, and a multidisciplinary individualized management strategy guided by ESC recommendations. Clinicians should maintain a high index of suspicion for aortitis in patients receiving G-CSF, and early use of CT and PET imaging is essential to guide management and prevent complications.

Learning points

- Clinicians should maintain a high index of suspicion for acute aortitis in patients receiving long-acting granulocyte colony-stimulating factor, like pegfilgrastim.
- Early use of computed tomography and positron emission tomography imaging is essential to guide management and prevent complications like dissections and aneurysms.

## Introduction

Oncologists commonly use PEGylated granulocyte colony-stimulating factor (G-CSF) to reduce chemotherapy-induced neutropenia by increasing stem cell mobilization.^1^ Even though pegfilgrastim is safe most of the time, it has rarely been associated with large-vessel vasculitis, in particular aortitis which may lead to fatal complications like aneurysm or dissection if not addressed right away.^2,3^ Here, we share the case of acute aortitis in response to pegfilgrastim which was effectively managed without glucocorticoids, relying on clear diagnostic investigations, specific imaging modalities, and adhering with ESC-endorsed vasculitis management principles.

## Summary figure

**Figure:**
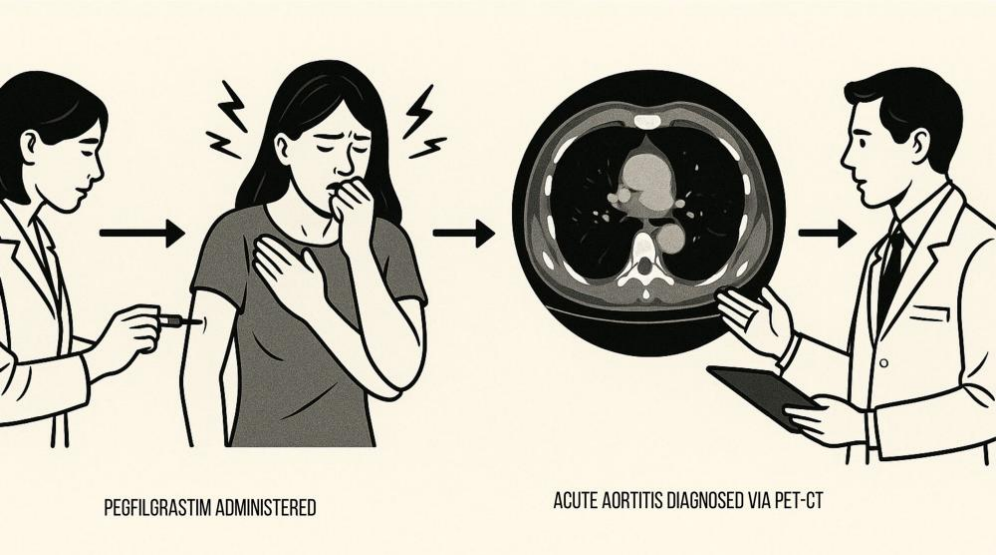


## Case presentation

### History of presentation

A 59-year-old woman with a history of pancreatic adenocarcinoma and prior breast cancer (treated at age 46 with mastectomy and chemotherapy) presented with fever, dry cough, and upper back pain that started 2 days following pegfilgrastim (Neulasta) administration for post-chemotherapy neutropenia support. The pancreatic adenocarcinoma was originally treated by systemic chemotherapy (modified FOLFIRINOX regimen) and an extensive non-curative surgical procedure. This was later followed by consolidative chemoradiation and received continuous palliative systemic chemotherapy with gemcitabine and nab-paclitaxel (Abraxane). She was treated with pegfilgrastim for the first time after her latest chemotherapy. Past medical history included steroid-induced diabetes mellitus, hypothyroidism, and an episode of remote pulmonary embolism.

At the presentation, her temperature was 38.1°C, with mild tachycardia but haemodynamic stability. Physical examination was unremarkable with no focal neurologic or cardiac findings, and no features suggestive of systemic vasculitis. The differential at the time included infectious aetiologies (bacterial, viral, fungal) and autoimmune conditions, given the non-specific systemic symptoms. Laboratory blood tests showed an increase in white blood cells of 15 000/µL, 80% neutrophils (normal: 4000 to 11 000/µL with 40%–60% neutrophils), and elevated C-reactive protein of 21.3 mg/dL (normal: <3 mg/dL) and erythrocyte sedimentation rate (ESR) of 65 mm/h (<30 mm/h for women over 50). The cultures of both blood and urine came back negative. The autoimmune workup which looked for ANA, ANCA, and RF was also negative.

**Figure 1:**
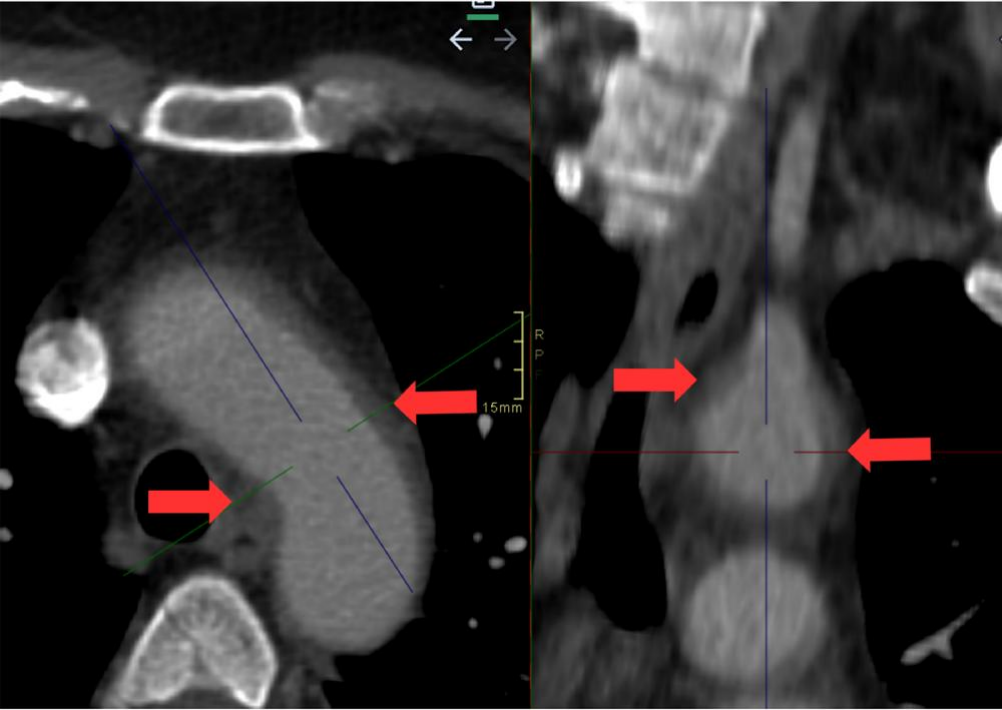
Contrast computed tomography: arrows demonstrated irregular wall thickening of the ascending and arch of the aorta with periaortic fat stranding.

**Figure 2:**
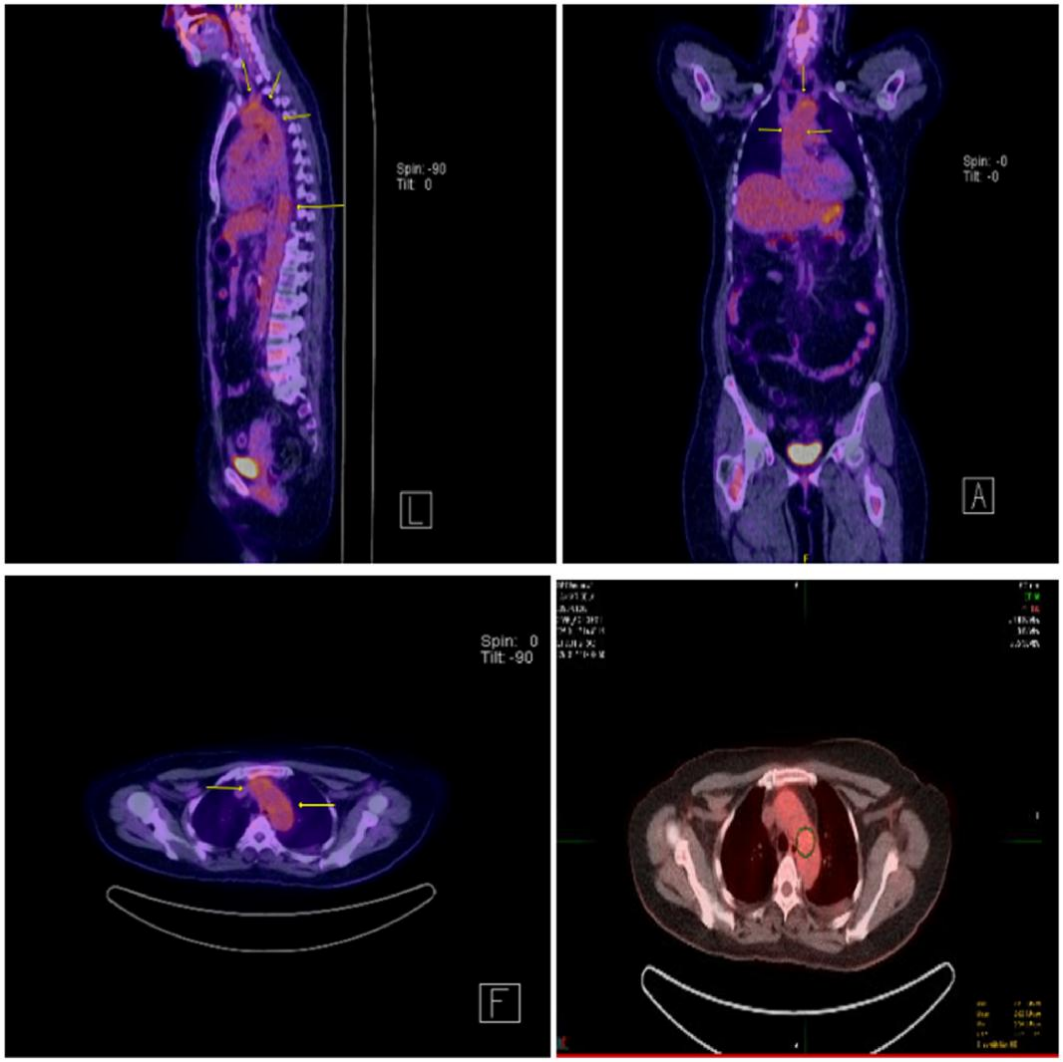
Positron emission tomography scan: arrows showing increased fluorodeoxyglucose uptake signifying aortic inflammation.

Contrast computed tomography (CT) demonstrated irregular wall thickening of the ascending and arch of the aorta with periaortic fat stranding; the aortic diameter was within normal limits (no aneurysm). No other vessels were involved (*Figure 1*). Positron emission tomography scan further demonstrated increased fluorodeoxyglucose (FDG) uptake in the ascending aorta and aortic arch with SUV max 3.5, greater than hepatic uptake, corresponding to grade II/III inflammation per current PET vasculitis criteria (*Figure 2*). Subsequently, a QuantiFERON-TB Gold test, a Rapid Plasma Reagin (RPR) test for syphilis and a Coxiella burnetii serology ruled out other possible causes of aortitis.

With both infectious causes and autoimmune disorders ruled out and since aortitis occurred after pegfilgrastim administration, G-CSF-induced aortitis was the probable diagnosis. Considering her prior history of steroid-induced diabetes and its complications, the multidisciplinary team (cardiology, rheumatology, oncology, and infectious disease) opted for cessation of Pegfilgrastim and conservative monitoring without corticosteroids.

**Figure 3:**
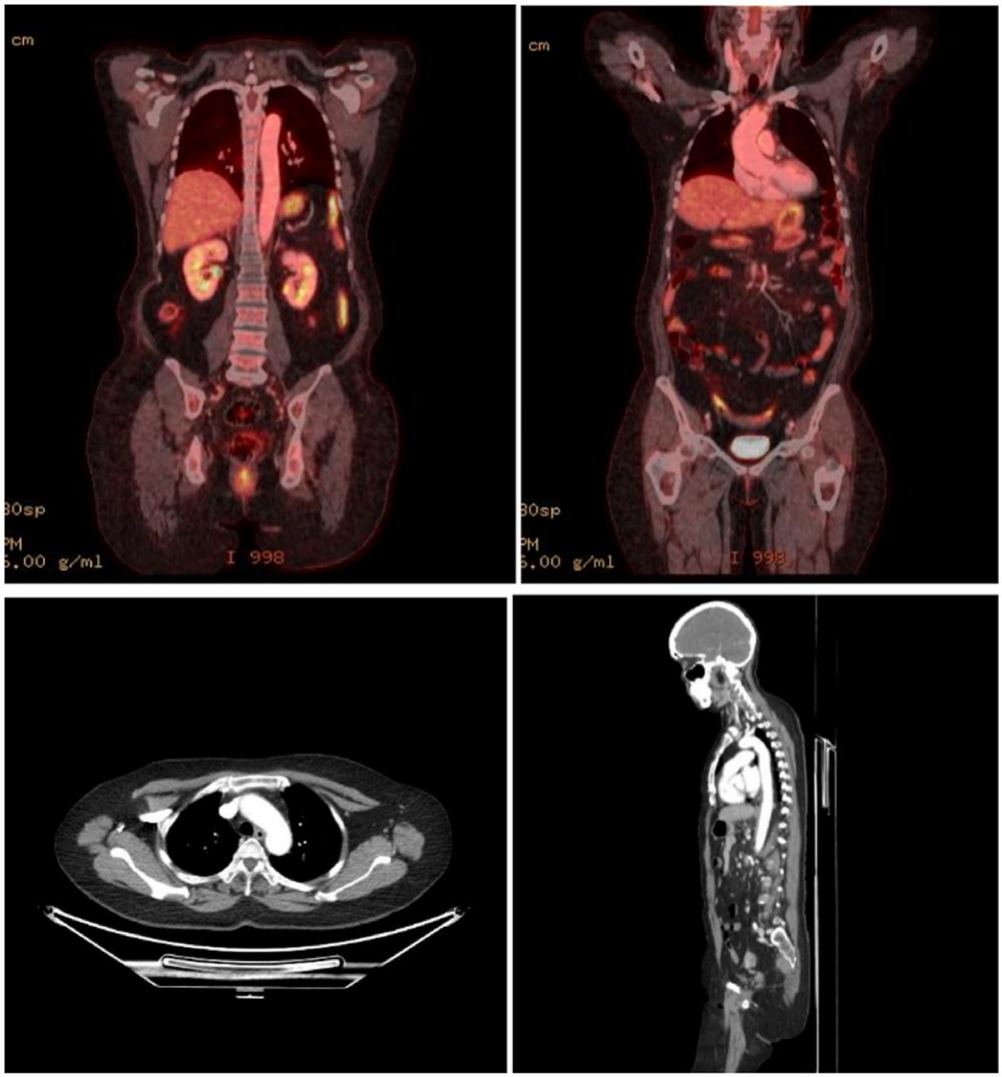
Post-symptom resolution positron emission tomography and computed tomography showing inflammation resolution.

The patient got frequent clinical and laboratory check-ups. In the following weeks, her symptoms went away completely and both her C-reactive protein and ESR returned to normal (1.5 mg/dL and 12 mm/h, respectively). A follow-up PET-CT in 2 months showed that all vascular FDG uptake and inflammation near the aorta had resolved (*Figure 3*).

## Discussion

### Pathophysiology: cytokine storm and endothelial injury

It is incompletely understood how G-CSF causes aortitis but presumably offers an immune-mediated vascular injury. The inflammatory activity resulting in the release of neutrophils and cytokines, such as IL-6, TNF-alpha, and IL-1-beta, followed by G-CSF, leaked into the system contributing to a cytokine storm.^4,5^

Further, G-CSF can upgrade the supplementary of ICAM-1, VCAM-1, and E-selectin through JAK/STAT and MAPK pathways to stimulate the endothelial cells.^4^ It improves leukocyte adhesion and transmigration that may result in injury to vessel walls and granulomatous infiltration, which is reported in histopathologic analysis of G-CSF induced vasculitis.^4,5^

### Rarity and risk factors

The condition of aortitis due to pegfilgrastim is not rare and is approximated at 0.3%–0.7% among the cancer patients undergoing pegfilgrastim (G-CSF).^6,7^ Almost all the reported cases happened during the past decade and can probably be explained by increasing awareness and greater usage of long-acting forms of G-CSF.^8^ The analysis of 72 cases revealed a mean age of the patient to be around 62 years old with 80% of the cases falling among women—most of them being breast cancer or gynaecological malignancies.^9^

The predisposition of populations of East Asians and senior women may be disease-related and may be due to genetic factors or pharmacologic reasons.^9,10^ Taxanes and cyclophosphamide—which is used in our patient—are common chemo medication reported with this complication.^6,10^ These identified risk factors coincide with the demographic and clinical profile that our patient would present, which incorporates the use of dose-dense chemotherapy.

It is important that clinicians should be cautious in the assessment of febrile neutropenia patients who are resistant to antibiotics since aortitis caused by drugs could be recurrent and exhibit similar characteristics as infectious or inflammatory disorders.^8^

### Causality and temporal association

Drug-induced aortitis is uncommon, yet G-CSF (and, in particular, pegfilgrastim) drugs have increasingly been reported as possible precipitants. Around 10 days after the injection, during our case, the development of fever and pain in the back corresponds to previous research in which aortitis is most likely to occur within 1–2 weeks following the injection of pegfilgrastim.^6,8^ The average onset was 13.4 days post-dose (range 1–38), in many cases occurring following the small initial number of injections.^6^ A causal relationship is supported by the strong temporal association in combination with the disappearance of symptoms once the drug is withdrawn. Conversely, the cases of others re-challenged with pegfilgrastim have resulted in its recurrence.^6,7^

Other possible causes were systematically ruled out. Cultures of blood and urine were negative, and serological titres were not outstanding to tuberculosis, syphilis, and Q fever. Autoimmune vasculitis giant cell arteritis (GCA) and Takayasu arteritis were unlikely as there was no obvious clinical history of those diseases (e.g. cranial symptoms or limbs calculation) and ANA, ANCA, and RF tests were negative.^8^ It was determined that IgG4-related aortitis would be excluded by having a normal serum IgG4. Without other explanations and with this relatively strict timing consideration around the use of pegfilgrastim, a diagnosis of drug-induced large-vessel aortitis was made.

### Imaging and positron emission tomography standardized uptake value grading

The imaging technique is central in diagnosing large-vessel vasculitis. Positron emission tomography/computed tomography offers semi quantitative imaging parameters in terms of standardized uptake value (SUV). SUVmax is a quantitative measure of the inflammatory tag which is the voxel with the greatest uptake in the area of interest and is reproducible.^11^

The visual grading of FDG uptake is scored in the scale of 03:

0: Uptake not seen (=mediastinal blood pool)

1: Low intake (<liver)

2: Medium uptake (=liver)

3: High uptake (>liver)^11^

An aortic SUVmax reading in our patient of 3.5 (greater than that of the liver) was a Grade 3 inflammation meaning that there was active vasculitis.

### Management: corticosteroids vs. conservative approach

Though corticosteroids are the common way of treating G-CSF induced aortitis, spontaneous recovery, without immunosuppression, has been reported. Comorbidities and early indication of clinical improvement informed the decision not to apply steroids to our patient.

More recent series have indicated that 30%–40% of patients could potentially recover without any corticosteroids, and the results were similar to those who do not receive steroid treatment.^7,9^ Ito *et al*. listed mild or self-limiting symptoms as possible cases when conservative and watchful approach can be adequate.^5^ The fever and inflammatory markers improved in our patient prior to any immunosuppressive agent which advocates this plan of action. In severe or refractory cases, however, corticosteroids are necessary.

### Differential diagnosis

A multifaceted diagnostic workup is important in ruling out other causes of aortitis. Aortitis due to infectious causes would likely show focal aneurysmal changes or positive cultures, neither of which are present here, ruling out syphilitic aortitis or mycotic aneurysm.^3,8^ Clinical or serological evidence did not support large-vessel vasculitides such as Takayasu arteritis (a more common vasculitis in patients < 50 years of age) or GCA (cranial symptoms are associated with this vasculitis).^8^

History, imaging, and laboratory values made it less likely that vasculitis was due to IgG4-related disease or radiation. This widespread exclusion of the mimics bolsters the possibility of drug-induced diagnosis. All these are mentioned in the subsection of the differential diagnoses and justified based on the existing literature.^8,10^

### Patient perspective

**‘**I was very frightened by the sudden upper back pain and fever. It was a relief when the scans showed the aorta was inflamed but getting better. I am grateful that the doctors found the cause and monitored me without extra medications.’

## Lead author biography


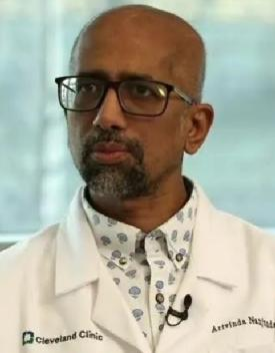


Dr Nanjundappa is an interventional cardiologist in the Invasive and Interventional Cardiology Section of the Robert and Suzanne Tomsich Department of Cardiovascular Medicine, Cleveland Clinic. Dr Nanjundappa is an active researcher and has been a principal investigator or co-investigator for more than 35 clinical research trials, including Medtronic’s Symplicity Spyral Renal Denervation (RDN) system. He has delivered invited lectures at more than 100 local, national, and international conferences and has presented abstracts at more than a dozen symposia. In addition, he has authored more than 125 publications including peer-reviewed articles, invited publications, and editorials, as well as more than 15 book chapters.

## Data Availability

Not applicable (no new data sets generated).

## Notes

**Consent:** The authors confirm that written consent for submission and publication of this case report including images and associated text has been obtained from the patient, in line with the COPE guidance.
